# Early Integration of Palliative Care for Patients Receiving Systemic Immunotherapy for Renal Cell Carcinoma

**DOI:** 10.33696/cancerimmunol.5.072

**Published:** 2023

**Authors:** Megan Stout, Akshay Thaper, Vivien Xu, Eric A. Singer, Biren Saraiya

**Affiliations:** 1 Division of Urologic Oncology, The Ohio State University Comprehensive Cancer Center, USA; 2 Rutgers Robert Wood Johnson Medical School, USA; 3 Division of Medical Oncology, Rutgers Cancer Institute of New Jersey, USA

## Introduction to Palliative Care

Palliative care (PC) aims to prioritize the quality of life and comfort of those with complex and terminal illnesses through the physical, psychological, social, and spiritual needs of the individual. Palliative care can support individuals from the day of their cancer diagnosis through end-of-life and bereavement [1,2]. In cancer care, when the primary oncology team addresses patients’ symptoms and helps with complex decision making, it is called primary palliative care. When the primary oncology team seeks help of expert palliative care teams for symptom management or in decision making, it is called secondary palliative care and when the expert palliative care team assumes the role of primary attending to address these needs, it is called tertiary palliative care [3,4]. Additionally, palliative care services may be found embedded in outpatient oncology clinics or as free-standing clinics, as hospice care, or via telehealth [5,6].

In practice, effective communication and trust-building are the cornerstones of delivering patient centered care, including palliative care. To overcome common shortcomings encountered during palliative care discussions, communication tools have been developed to effectively address these gaps and help patients make personally meaningful decisions based on tangible outcomes that are tailored to their specific circumstances [1,7–10]. For example, one framework was created to ascertain levels of cultural influence on palliative care, addressing the patient’s Attitudes, Beliefs, Context, Decision-making style, and Environment (ABCDE) [10]. A recent adaptation of the Best Case/Worst Case framework uses visual aids depicting the best, worst, and most likely scenarios to encourage patient understanding and autonomy in decision-making [8]. With effective communication, integration of palliative care early in the cancer journey empowers patients to evaluate their options in the context of their values and prioritize their needs, which may include supportive or life-extending measures, as well as crisis prevention [11].

While clinical trials have shown that early integration of PC is correlated with improved pain and symptom control, better quality of life, reduced hospitalizations and survival for individuals with advanced cancers (including lung, breast, gastrointestinal, and biliary), the literature also suggests that there is a dearth of early palliative care integration for individuals with genitourinary cancers [5,12–23]. This commentary will report current evidence for palliative care integration in oncologic care, track palliative care interventions in genitourinary malignancies with special focus on palliative care integration for those on systemic immunotherapy for renal cell carcinoma, and offer approaches to address the limitations associated with early integration of palliative care services for individuals with renal cell carcinoma (RCC).

## Current Evidence for Palliative Care Integration in Oncologic Care

There is substantial evidence regarding the positive beneficial effects of early integration of PC within the field of oncology. Several large single-center and multicenter randomized trials of early integration of secondary or subspecialty PC teams in routine oncologic care have shown improvements in patient overall mood and quality of life (QOL), as well as the potential to improve patient survival [16,17,24]. Furthermore, randomized control studies and retrospective data suggests overall survival (OS) and QOL benefits to those receiving early outpatient palliative care as well as inpatient palliative interventions [17,25,26]. In addition to benefit to patients, studies also highlight positive impact of palliative care on caregiver satisfaction and healthcare-related costs [27]. These beneficial effects of palliative care warranted an update to the American Society of Clinical Oncology clinical practice guideline in 2017, with the key recommendation being that patients should be referred to interdisciplinary palliative care teams for secondary PC early in their disease course in addition to the primary palliative care delivered by the primary oncology team [28].

Despite the supporting evidence, many cancer patients are still not receiving early palliative care. For example, only 8% of lung cancer patients in the United States see a PC specialist, and these visits are often near death to address end-of-life issues [29[. At an established palliative care cancer program, only 32% of decedent cancer patients received a palliative care consultation in 2010–2012, with only 10% of patients receiving early consultations; 82% of the patients that received PC intervention late in their disease trajectory did so for endof-life hospitalization alone [30].

## Current Palliative Care Interventions in Genitourinary Malignancies

Despite representing nearly 25% of new cancer diagnoses, with over 350,000 new cases diagnosed each year in the US, few genitourinary (GU) patients have been included in the above palliative care trials [31] The Advanced Prostate Cancer American Urologic Association/American Society for Radiation Oncology/Society of Urologic Oncology 2020 guideline encourages palliative care involvement in managing symptoms such as pain, urinary symptoms, and sexual function, as well as side effects of treatment [32]. Currently, studies have shown a mean 16.6% delivery rate of inpatient palliative intervention in patients with metastatic bladder, prostate and kidney cancer, with more frequent utilization in patients who present with the most advanced disease [31]. The same study found that Black and Hispanic patients with prostate or kidney cancer were 29% less likely to receive palliative intervention than Caucasians [33]. Older patients were also less likely to receive palliative interventions, possibly due to difficulty to distinguish between frailty and disease process [33,34]. In renal cell carcinoma, it was found that use of palliative care has minimally increased from 17% to 20% for Stage IV RCC from 2004–2014, with only 18.8% of the analytic cohort receiving intervention [35].

## Palliative Care Integration in Advanced Renal Cell Carcinoma (RCC)

RCC makes up approximately 90% of current kidney cancer cases [36]. Approximately 20–40% of patients with localized disease eventually develop distant metastasis after nephrectomy and additionally, a third of patients are diagnosed with metastatic disease on presentation [37]. In the past two decades, improved understanding of RCC cancer biology has led to approval of vascular endothelial growth factor (VEGF) pathway drugs including tyrosine kinase inhibitors (TKI) and the mammalian target of rapamycin (mTOR) inhibitors and more recently immune checkpoint inhibitors (ICI) and combination of these agents. Immune checkpoint inhibitors as well as targeted therapies have improved patient outcomes and represent the mainstay of treatment for advanced untreated metastatic RCC [38]. Efficacy of these agents have been demonstrated in first-, second-, and third-line settings [39]. These discoveries have improved patient outcomes such as improvement in patient survival as well as long term remissions or potential cure of metastatic renal cell cancer [38–41]. All treatments for metastatic RCC have been primarily studied in clear cell RCC, which can substantially differ in non-clear cell histologic settings, though several clinical trials are underway to develop therapies specific to non-clear cell tumors [42].

In the early disease setting where surgery is curative, approximately 20–40% of patients will develop recurrence or distant metastasis after surgery. Multiple trials have explored the potential benefit of oral tyrosine kinase inhibitor systemic therapies following surgical extirpation for high-risk localized RCC while showing progression free survival (PFS) benefit, all have failed to show an OS benefit [43]. Most recently approved, an immune checkpoint inhibitor, pembrolizumab, has also shown benefit of decreasing recurrence rates in patients with high risk of recurrence (patients with pathological findings of: pT2 stage with nuclear grade 4 or sarcomatoid differentiation, ≥ pT3, nodal disease, or need for nephrectomy and metastasectomy) with pending OS data [41,44].

Treatment paradigms for RCC that were once concrete in the setting of localized versus metastatic disease with curative surgical resection and palliative systemic therapies as two possibilities of intervention, respectively. With recent advances, the paradigm has now become more nuanced. Through the approval of adjuvant therapies including immunotherapy, systemic interventions are used in curative setting and PC can benefit patients in this setting as well. Newer combination systemic therapies (doublets and triplets) for patients with metastatic disease show long term complete response and possible cure in approximately 10% of cases. Thus, integration of early PC is more important and necessary than ever.

**Figure 1.**
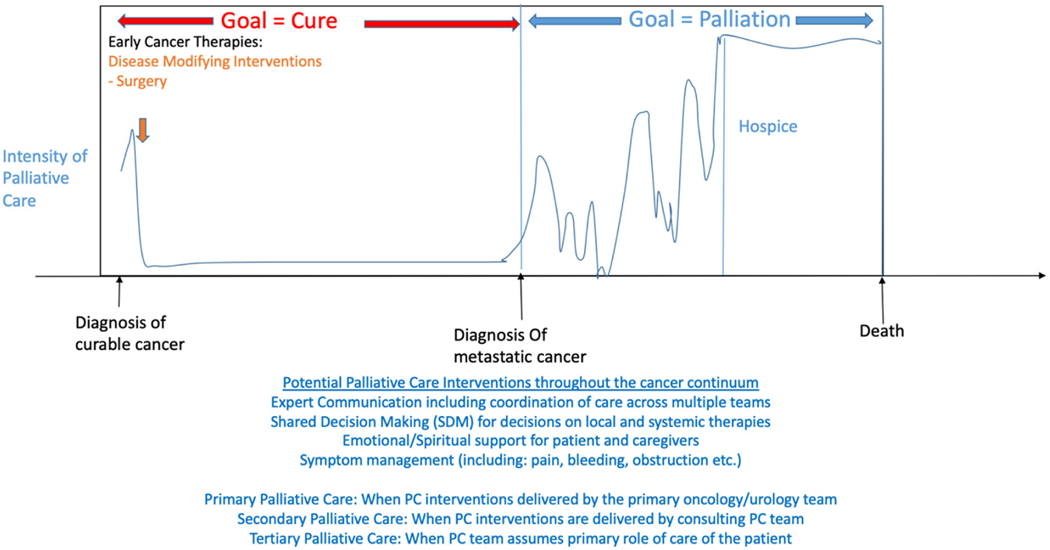
Integration of Palliative Care Interventions Throughout The Course of Management of Kidney Cancer (Including Systemic Immunotherapy Use) from Diagnosis and Curative Treatment to Palliative Focus and Hospice [45].

As shown in Figure 1 and Table 1, primary PC interventions defined can be applied in curative as well as palliative setting, for not only local but also in a systemic symptom management. Local interventions such as stents to alleviate obstruction or bleeding; help in making a patient centered shared decision regarding curative surgery are examples of primary palliative care intervention delivered by urologists [45]. In palliative setting, primary palliative care can be delivered by medical, radiation and urologist oncologist. Systemic interventions by medical oncologists for treatment symptoms from cancer including pain or adverse effects of systemic therapies including immunotherapy such as fatigue, enteritis, pneumonitis, rashes, hypertension related symptoms in accordance with guidelines are examples of primary PC [46–51]. Radiation for painful metastasis are examples of primary PC delivered by radiation oncologists.

Opportunities for secondary palliative care interventions can also be thought of in the context of curative versus noncurative settings.45 An example of the integration of palliative care in these contextual settings throughout the RCC disease progression is seen in Figure 1 [45]. In the curative setting, goals of care for the cancer in mind is eradication of the cancer – which may require a mix of surgical, radiation, systemic therapies, or even a combination thereof. Effects of these therapies can include not only post-operative physical but also psychological or emotional symptoms. Palliative care teams can work in conjunction with these therapy options to improve overall patient symptoms including pain or function [52]. In the non-curative, or palliative setting, goals of care include prolonging the quality of life of the individual. This requires patient input regarding how they would like to live their life – with palliative care focusing again on emotional/spiritual, pain, and symptom relief from cancer or cancer therapies.

Despite the number of patients undergoing treatment that may benefit from supportive care interventions, PC usage for RCC remains poorly integrated [35]. An analysis of the National Cancer Database (NCDB) found that less than 20% of patients with advanced RCC are coded to have received palliative care, with increased comorbidities, insurance status, higher education status, facility location, and care at a comprehensive cancer program significantly increased the likelihood of PC intervention use [35]. This is also consistent with NCDB data for patients with metastatic prostate, bladder, and kidney cancer from 2004–2013: only 19.9% of kidney cancer patients utilized palliative care interventions [33]. With new therapy options in treatment for advanced and metastatic RCC, it is clear that there remains a need for an integrated approach to the initiation of PC and further patient and provider education regarding its benefits in this setting.

## Obstacles or Limitations to Palliative Care Integration for Those on Therapy for RCC

Despite the numerous benefits of early integration of palliative integration for those with advanced RCC, potential obstacles and challenges exist preventing this integration which is derived from similar experiences across the entire cancer care continuum. Only about 14% of patients who need palliative care receive it according to the World Health Organization [53]. Barriers to integration of care can be divided into structural, provider, and patient-family barriers [45].

One recent survey demonstrated that of United States cancer centers, 95% of National Cancer Institute (NCI)-designated institutions had outpatient palliative care programs versus only 40% of non-NCI centers [54]. Resources remain limited in rural or other areas outside of cancer center networks limiting patient access to providers with palliative care expertise [55]. An additional study highlighted infrastructural limitations to palliative integration with advanced RCC finding that those receiving care at a comprehensive community cancer program or integrated cancer network were more likely to receive palliative intervention [40]. There also remains a critical palliative workforce shortage. The gap between the current supply of hospice and palliative care physicians and the hypothetical demand to reach mature staffing levels is approximately 6,000–18,000 individual physicians [56].

Provider barriers for early integration of palliative care includes lack of understanding of palliative care and its assumed association with hospice and inevitably death, lack of exposure or training beyond residency, and lack of understanding of local resources and available at their institution [57,58]. Additionally, many clinicians equate palliative care with end-of-life care, which likely leads to possibly fewer and later referrals [24].

Patient barriers include lack of understanding of the definition of palliative care, lack of knowledge of available resources, cost, patient-provider emotions, or inaccurate prognostic interpretation of their overall disease state [45]. A national survey of 800 adults which found that 70% of participants were not at all knowledgeable about palliative care [59]. In a report evaluating expression of emotion in both patients and their families undergoing discussions regarding palliative care, nearly 70% of conversations consisted of some form of emotional distress, most commonly anxiety and fear followed by sadness, anger, or frustration [60].

Oncologists themselves are not devoid of emotion – the impact on providers can be heightened when they have established a long-term patient-physician relationship throughout the course of a patient’s cancer diagnosis and treatment. One study demonstrated that the longer an oncologist had known a patient, the less accurate their prediction and disclosure of the prognosis became [61]. Furthermore, only 38% of patients reported ever having discussed longevity expectations with their oncologist, with only 5% of patients having an accurate understanding about the incurability of their disease or timeline of decline [62].

These challenges or limitations are even more apparent in the era of immunotherapy use in the treatment of RCC. As previously mentioned, immunotherapy can provide a potential for cure in a cohort that were previously thought to have incurable disease. In a study of those with advanced GU cancers, the authors found that anxiety scores were actually lower in patients with accurate understanding of the expected outcomes of immunotherapy and their disease course [63].

## Potential Approaches to Increased Integration for Providers and Future Directions

Overall, the steps for early integration of palliative care for patients with renal cell carcinoma starts with evaluation of patient function, disease stage and eliciting patient goals, values and preferences. In addition to addressing physical symptoms of cancer and cancer treatment, optimal care involves Identification and addressing emotional, spiritual or practical needs. For those symptoms not able to be addressed by the primary team, other members of the teams including PC team can be consulted. There needs to be coordination of care with the patient’s primary oncology team and other members of the healthcare team, such as nurses, social workers, and chaplains, to ensure that all aspects of the patient’s needs are addressed. Supportive interventions may be offered such as counseling or therapy, to help the patient cope with the emotional impact of the cancer and its treatment. Education of the patient and their family about the disease and treatment, prognosis, and realistic goals of care, to help with shared decision making. Finally, regular assessment of the patient’s needs and adjusting the care plan is necessary to ensure that the patient’s symptoms are well-managed, and their overall well-being is supported throughout their treatment trajectory. The benefits of early integration of palliative care can include improved symptom management, better communication and coordination of care, and greater support for patients and their families throughout the cancer journey.

Despite the points highlighted on how to approach this integration, there remains limited data on palliative intervention in the setting of advanced RCC, we present opportunities to overcome possible limitations or barriers to integration in routine oncologic care. In terms of lack of a supportive infrastructure or access to care, with one evident barrier being patient transportation for frequent palliative care visits, the increased acceptance of telemedicine with enhanced focus on nursing phone follow-ups can overcome these structural barriers [14,64].

Provider barriers can be addressed with improved understanding of PC interventions as well as structured exposure and education to these processes. A survey administered to oncology fellows in 2011 demonstrated that only 26% had completed a palliative care rotation, with only 32% of fellows receiving explicit education on assessing and managing depression at end of life [65]. For those that were exposed to a palliative care rotation, fellows reported improved knowledge and attitudes in all palliative integration domains [66]. No formal palliative care rotation or curriculum is required for urology residents at this time, and remains an area of future study and need [67]. In order to emphasize various steps of intervention that can be implemented by a provider enabling palliative care, we present specific tasks that primary or sub-specialty care teams can enact to address specific patient needs (Table 1) [45]. Example patient needs are provided for if the patient is in the localized or metastatic disease state, and provider skills/skillsets required to enact these interventions for the patient are highlighted.

In terms of improved provider skills and understanding in order to mitigate PC discussions, one must perform a self-assessment of the individual skillsets of any advanced RCC provider such as a urologist, medical oncologist, or radiation oncologist that are part of the primary PC team. Based on the self-assessment of the primary oncology team members, in addition to the primary palliative care they provide, they must refer to secondary palliative care when appropriate. In addition to the development of basic communication skills, there are specific skills in palliative care delivery including symptom assessment and management, gauging of emotional symptoms, and patient centered discussions on decision-making. We must also consider the multidisciplinary aspect of palliative care administration outside of oncologic providers alone. A study identified the surgeon’s role outside of clinical and operative decision-making, stating that surgeons also work towards understanding patient and family goals, facilitating challenging and tense discussions, and fostering shared decision making. Data supports that early palliative care involvement alongside surgical treatment facilitates goalconcordant decision making, improves patient emotional and spiritual well-being, and enhances overall quality of life [68].

Finally, patient-provider barriers can be overcome with standardization of disease prognosis and decision-making discussions, as well as interval assessment of patient understanding of their treatment course. One group developed a preliminary measure for patient prognostic understanding of their own condition, Prognostic Understanding Perceptions Scale (PUPS), which has been utilized for those with advanced cancers in order to further facilitate patient-physician discussions [69]. This, along with tools such as Best Case/Worst Case visual aids may effectively address gaps in communication barriers and help patients make personally meaningful decisions based on tangible outcomes that are tailored to their specific circumstances [8].

Despite the evidence of survival benefit demonstrated by those utilizing early integration of palliative care use, studies do not definitively demonstrate why these patients may have benefited. One focused area of study could include the improved patient understanding of their disease status and shared-decision making models – patients not only need to understand their goals, values, and preferences, but also their treatment options including the risks and benefits of each. Determining more focused goals of providers in these discussions in the interplay of oncologic teams and palliative care physicians as primary team members or in the consultant role, can guide future research into how these mechanisms are best applied.

With the current advancements in immunotherapy use in the setting of advanced RCC, traditional definitions of curative and palliative disease states are blurred. This enhances the need for comprehensive and systematic integration of palliative care. With increased integration, comes enhanced awareness of not only structural but patient-provider barriers that once identified can be overcome with improved communication and multi-disciplinary team efforts. Increased areas of study within primary teams for RCC with focus on shared decision making and patient centered care may help develop improved interventions for RCC and across the cancer care continuum.

## Figures and Tables

**Table 1.** Example of patient symptoms that may require palliation for a patient with advanced RCC [45].

Example Patient Needs in Localized Disease	Provider Skills Required
Understanding of Diagnosis	Medical knowledge; ability to effectively communicate knowledge
Informed Decision Making	Ability to elicit patients’ desires to utilize in shared decision making
Goal-Aligned Treatment	Ability to assess patient goals and discuss how each treatment option could impact patient goals
Post-Operative Pain	Familiarity and knowledge of pharmacological and non-pharmacological modalities to alleviate pain
Surveillance-Related Anxiety	Ascertain and evaluate the key sources of patient anxiety, Communicate and interpret test results that are meaningful to patients
Family Discussion / Support	Facilitate patient and caregiver communication about diagnosis and prognosis
Example Patient Needs in Metastatic Disease	Provider Skills Required
Emotional Symptoms – Anxiety, Depression etc.	Evaluate and address patient’s emotional symptoms due to cancer, cancer treatment
Prognosis Understanding	Communication Skills to elicit patient understanding and then providing patient information in empathic manner
Uncertainty / Life Expectancy	Elicit and manage emotions related to uncertainty Plan for uncertainty
Goals of Care Discussion/Shared Decision Making	Elicit patient and family perspective Engage patient and family in discussion about patient’s goals Provide information on various options and help devise a plan that maximized likelihood of meeting patient’s goals
Physical Symptoms or Systemic Therapy Adverse Effects – Pain, Nausea, rash, diarrhea, shortness of breath, fatigue etc.	Knowledge on evaluation and management of each of the symptoms Management of various immunotherapy agents Multi-disciplinary discussions Seek assistance when needed

## Abbreviations

PC: Palliative Care
ABCDE: Attitudes, Beliefs, Context, Decision-making style, and Environment
RCC: Renal Cell Carcinoma
QoL: Quality of Life
OS: Overall Survival
GU: Genitourinary
VEGF: Vascular Endothelial Growth Factor
TKI: Tyrosine Kinase Inhibitors
mTOR: Mammalian Target of Rapamycin
ICI: Immune Checkpoint Inhibitors
PUPS: Prognostic Understanding Perceptions Scale
PFS: Progression Free Survival
NCDB: National Cancer Database
NCI: National Cancer Institute
